# Risk of transmission of sporadic Creutzfeldt-Jakob disease by surgical procedures: systematic reviews and quality of evidence

**DOI:** 10.2807/1560-7917.ES.2017.22.43.16-00806

**Published:** 2017-10-26

**Authors:** Fernando J García López, María Ruiz-Tovar, Javier Almazán-Isla, Enrique Alcalde-Cabero, Miguel Calero, Jesús de Pedro-Cuesta

**Affiliations:** 1 National Epidemiology Centre, Carlos III Institute of Health, Madrid, Spain; 2 Center for Biomedical Research in Neurodegenerative Diseases (CIBERNED), Madrid, Spain; 3 Chronic Disease Programme, Carlos III Institute of Health, Madrid, Spain; 4 Alzheimer Disease Research Unit, CIEN Foundation, Queen Sofia Foundation Alzheimer Centre, Madrid, Spain

**Keywords:** Transmissible spongiform encephalopathies, CJD, Creutzfeldt-Jakob disease, Surgery, Systematic review, Case-control studies, Neurosurgery

## Abstract

Background: Sporadic Creutzfeldt–Jakob disease (sCJD) is potentially transmissible to humans. Objective: This study aimed to summarise and rate the quality of the evidence of the association between surgery and sCJD. Design and methods: Firstly, we conducted systematic reviews and meta-analyses of case–control studies with major surgical procedures as exposures under study. To assess quality of evidence, we used the Grading of Recommendations, Assessment, Development and Evaluations (GRADE) approach. Secondly, we conducted a systematic review of sCJD case reports after sharing neurosurgical instruments. Results: Thirteen case–control studies met the inclusion criteria for the systematic review of case–control studies. sCJD was positively associated with heart surgery, heart and vascular surgery and eye surgery, negatively associated with tonsillectomy and appendectomy, and not associated with neurosurgery or unspecified major surgery. The overall quality of evidence was rated as very low. A single case–control study with a low risk of bias found a strong association between surgery conducted more than 20 years before disease onset and sCJD. Seven cases were described as potentially transmitted by reused neurosurgical instruments. Conclusion: The association between surgery and sCJD remains uncertain. Measures currently recommended for preventing sCJD transmission should be strongly maintained. Future studies should focus on the potential association between sCJD and surgery undergone a long time previously.

## Introduction

Creutzfeldt–Jakob disease (CJD) is a neurodegenerative disorder with deposition of a pathological isoform (transmissible spongiform encephalopathy-associated prion protein—PrP^TSE^) of the normal cellular prion protein (PrP^C^). CJD exists in three forms: sporadic (sCJD), acquired – either variant (vCJD) or accidentally transmitted (atCJD) – and caused by mutations in the gene encoding PrP – here denoted as genetic CJD (gCJD) [1]. All of these can be experimentally transmitted to mammals by diverse procedures, e.g. directly to brain, eye or peritoneum or by corneal instillation [2]. Surgical transmission resulting in atCJD has been reported after use of cadaveric dura mater grafts, treatment with human pituitary growth or human gonadotropin hormones, corneal transplant from a donor diagnosed with CJD and exposure to neurosurgical instruments previously used on a case of human prion disease [3]. In addition, experimental evidence in animal models suggests that both vCJD and sCJD may be surgically transmitted by routine procedures [4].

Since 1994, the presence of infectivity in several tissues and organs from patients with sCJD has been widely recognised. Brown et al. demonstrated infectivity in homogenates of brain, lung, eye, kidney and other tissue from patients diagnosed with sCJD [5]. The World Health Organization (WHO) *Tables on tissue infectivity distribution in transmissible spongiform encephalopathies* classify tissue into the following three types: tissue with high proven infectivity (PrP^TSE^ detected in humans), whether for vCJD or other transmissible spongiform encephalopathies, tissue with lower infectivity and tissue with no detected infectivity or PrP^TSE^ [6]. While epidemiological evidence for surgical transmission of sCJD by blood, dental treatment or endoscopic procedures is lacking, evidence for sCJD transmission has been considered limited owing to the high risk of bias in case–control studies addressing this subject [7]. Notwithstanding this lack of evidence, 14 of 16 representatives from European Union countries and Norway reported that their country had guidelines on the prevention of transmission of human transmissible spongiform encephalopathies in medical settings, such as quarantining of instruments until firm diagnosis of a suspected infective patient [8].

Two types of design have been used to study potential surgical transmission of sCJD, namely case–control studies and case reports focusing on neurosurgical procedures. The purpose of this study was to review and assess the evidence, across studies, of surgical transmission of sCJD for specific surgical procedures and for neurosurgical procedures in particular. This evidence should constitute a scientific basis for proposing specific public health recommendations and guidelines for the prevention of sCJD transmission in medical settings.

## Methods

We divided our study into two parts: firstly, we performed a systematic review of observational analytical studies (case–control and cohort) investigating the relationship between any surgical procedure and sCJD incidence; and secondly, we reviewed case reports on potential transmission of sCJD by non-disposable instruments reused after neurosurgical procedures conducted on patients with CJD.

### Systematic review of observational analytical studies

#### Objective and search strategy

Our objective was to conduct a systematic review of associations between surgery and sCJD. Our data source was MEDLINE for the period from 1946 to 1 March 2016. We used the search strategy of combining (prion diseases OR prions OR Creutzfeldt-Jakob syndrome) AND (cohort OR prospective OR longitudinal OR case–control). In addition, we conducted a manual search of the references of studies selected according to the criteria explained below, studies found in a previous systematic review [7], and studies of interest known to the authors but not included in the MEDLINE search.

#### Selection of studies, inclusion criteria and data-extraction

Three authors (JA-I, MR-T, FJGL) selected the studies based initially on their title and then on their abstracts. Any disagreements were resolved by discussion. Our inclusion criteria were the presence of two groups (exposed and non-exposed group for cohort studies, and case and control group for case–control studies), any major surgical procedure as exposure and an effect measure to assess risk (risk ratios in cohort studies and odds ratios in case–control studies). We excluded studies on minor surgery (in which exposures are not usually well-defined), studies with a latency period between surgery and sCJD onset of less than 1 year, and animal studies. Two authors (MR-T and FJGL) extracted data, with any disagreements being resolved by discussion.

#### Risk-of-bias analysis

To assess risk of bias in individual studies, we first used the Newcastle–Ottawa scale which was designed to assess the quality of non-randomised studies in meta-analysis [9]. With this scale, every study is judged on three broad bases, i.e. selection of study groups, comparability of groups and ascertainment of the exposure or outcome of interest for case–control or cohort studies, respectively. A higher score indicates a higher study quality, with a score range of 0 to 10 for both case–control and cohort studies [9]. Subsequently, we added one of the previously defined criteria [7] not taken into account in the Newcastle–Ottawa scale, control-sampling design, i.e. whether or not they were sampled concurrently with cases. In particular, we rated whether the predominant type of control sampling had been longitudinal (or mid-point) or at the end of the study. Two reviewers (MR-T, FJGL) assessed studies’ risk of bias.

#### Data synthesis

**Statistical synthesis of effect estimates**

To synthesise evidence, we grouped studies into categories defined by anatomical type of surgery (cardiovascular, tonsillectomy, appendectomy, gall bladder, eye, dental, neurosurgery and any other surgery) and by time between surgery and symptom onset. For meta-analysis, we chose from each study the effect measure between surgery and sCJD incidence obtained from the most adjusted statistical analysis. We used DerSimonian and Laird random-effects models [10] to summarise effect measures in meta-analysis within types of surgery.

**Heterogeneity**

We determined heterogeneity with Cochran’s chi-squared test (Cochran’s Q), quantified with the *I* ^2^ statistics (range: 0–100%), with interpretation of low, moderate and high heterogeneity corresponding to *I* ^2^ values < 25%, 25–50% and 50–75% [11], with statistically significant heterogeneity being set at a p value < 0.10.

**Sensitivity analysis**

We performed two sensitivity analyses for every meta-analysis. Firstly, we recalculated the pooled association, after removing every single study from the meta-analysis (‘leave one out’ approach) [12]. Secondly, we removed studies with Newcastle–Ottawa scale scores < 8 and studies with non-longitudinal control-sampling design and recalculated the summary association.

**Publication bias**

We used funnel plots and Egger’s tests (with a significance level set at 0.10) [13] to assess potential publication bias. We also applied the ‘trim and fill’ method [14] to estimate a new summary measure, taking the ‘missing’ studies into account. Although all three methods were used solely in meta-analyses having at least 10 studies, this procedure was also followed for meta-analyses with fewer studies if their Egger’s test suggested publication bias.

**Software**

Review Manager 5.3 (Nordic Cochrane Center, Cochrane Collaboration, Copenhagen, Denmark) was used to draw forest and funnel plots and perform statistical analyses. Stata 14.1 (StataCorp, College Station, TX) was used to perform sensitivity analyses and publication bias tests, with the commands ‘metan’, ‘metatrim’, ‘metafunnel’ and ‘hatred’.

#### Overall quality of evidence

To assess the quality of evidence, we used the Grading of Recommendations Assessment, Development and Evaluation (GRADE) system, a system designed to rate quality of evidence of risk and grade strength of recommendations in systematic reviews, health technology assessment and clinical practice guidelines [15,16]. The GRADE system provides four levels of evidence, i.e. high, moderate, low and very low, depending on the extent to which one can be confident that the true effect is close to the estimate of that effect. In our study, we rated the quality of evidence of every type of surgery using the information furnished by their respective contributing studies. Following the GRADE system procedure for observational studies, rating began by awarding a low quality level and then revising this upwards or downwards once the following eight categories had been considered: risk of bias, inconsistency, indirectness, imprecision, publication bias, large effect, dose response, and all plausible residual confounding. If risk of bias, inconsistency, indirectness or publication bias was present, the quality level was revised downwards. On the other hand, if there was a large effect measure, a dose response between exposure and effect or if all possible residual confounding would tend to increase the magnitude of the effect, then the quality level was revised upwards. The final quality level reflected the sum of all these potential factors [17].

### Systematic review of case reports on potential transmission of sCJD by instruments used in neurosurgical procedures

We conducted a literature search in MEDLINE to find paired-case reports linked by exposure to neurosurgical instruments with a potentially infective case and a presumable secondary case. The search strategy included the terms (case series OR case report) AND Creutzfeldt-Jakob AND neurosurgery. We also included references already known to the authors. From the papers so selected, information was obtained by MR-T and JPC on evidence of shared exposure to the same instruments, operation dates, sCJD diagnoses, as well as dates of clinical onset and death of potentially secondary cases.

## Results

### Systematic review of observational analytical studies

#### Literature flowchart

**Figure 1:**
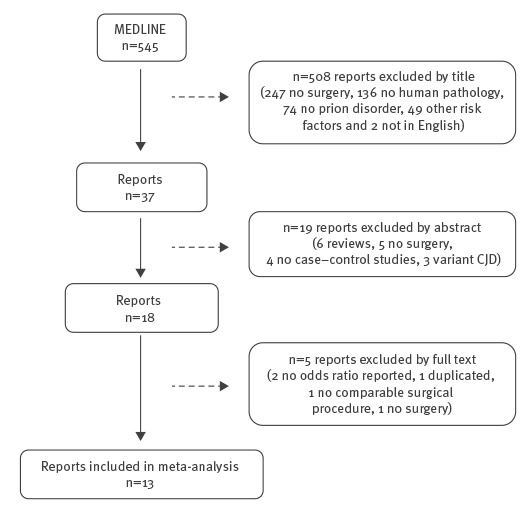
Flowchart of case–control studies assessing the association between surgery and sporadic Creutzfeldt–Jakob disease (n = 545)

Figure 1 shows the review flowchart. Of 545 references found in MEDLINE, 18 were retained after the rest had been discarded on the basis of the information drawn from their titles and abstracts.

CJD: Creutzfeldt–Jakob disease.

After reading the full text, a further five were excluded for not fulfilling the selection criteria (data provided by the authors on request). This left 13 studies, all case–control, valid for systematic review and meta-analysis purposes. Table 1 shows some characteristics of the studies included in the review.

**Table 1:** Characteristics of studies included in the systematic reviews of case–control studies assessing the association between surgery and sporadic Creutzfeldt–Jakob disease (n = 13)

Study	Country	Year	Type of surgery	Number of cases	Number of controls	NOS	Longitudinal control sampling
Harries-Jones et al, 1988 [28]	England and Wales	1980–84	Any surgery	122 (93 definite, 29 probable)	2 per case (184)	6	Yes
Collins et al, 1999 [18]	Australia	1970–77	Cardiovascular, tonsillectomy, appendectomy, gall bladder, eye, dental, any surgery	241 (151 definite, 90 probable)	More than 3 per case (784)	7	No
Laske et al, 1999 [29]	Germany	1997–98	Any surgery	37 (7 definite, 30 probable)	37	5	Yes
Zerr et al, 2000 [22]	6 European countries	1993–95	Tonsillectomy, appendectomy, gall bladder, eye, dental, neurological, any surgery	405 (199 definite, 206 probable)	405	5	Yes
Nakamura et al, 2000 [27]	Japan	1996–99	Appendectomy, any surgery	52 of 83 reported CJD cases	102	4	Yes
Ward et al, 2002 [23]	4 European countries	1993–95	Tonsillectomy, appendectomy, gall bladder, eye, neurological, any surgery	326 (169 definite, 157 probable)	326	6	No
Ward et al, 2008 [20]	United Kingdom	1998–2006	Cardiovascular, tonsillectomy, appendectomy, eye, dental, neurological, any surgery	431 (298 definite, 133 probable), Median age: 51 years	454	8	Yes
Mahíllo-Fernández et al, 2008 [19]	Denmark and Sweden	1994–2003 (Denmark), 1987–2002 (Sweden)	Cardiovascular	167 (113 definite, 54 probable)	835 matched, 2,224 unmatched	10	Yes
Hamaguchi et al, 2009 [24]	Japan	1999–2008	Eye, neurological, any surgery	753 definite or probable	210	3	Yes
Ruegger et al, 2009 [25]	Switzerland	2001–2004	Eye, appendectomy, dental, neurological, any surgery	69 cases 61 definite 8 probable	224 matched	6	No
De Pedro-Cuesta et al, 2011 [21]	Denmark and Sweden	1994–2003 (Denmark), 1987–2002 (Sweden)	Cardiovascular	167 (113 definite, 54 probable)	835 matched, 2,224 unmatched	10	Yes
Puopolo et al, 2011 [30]	Italy	1993–2008	Any surgery	741 (563 definite, 178 probable)	482	6	Yes
Surgery time predating diagnosis							
De Pedro-Cuesta et al, 2014 [26]	Denmark and Sweden	1994–2003 (Denmark), 1987–2002 (Sweden)	Time lag between surgery and disease onset	167 (113 definite, 54 probable)	835 matched, 2,224 unmatched	10	Yes

CJD: Creutzfeldt–Jakob disease; NOS: Newcastle–Ottawa scale.

#### Types of surgery positively related to sCJD

**Heart surgery**

**Figure 2:**
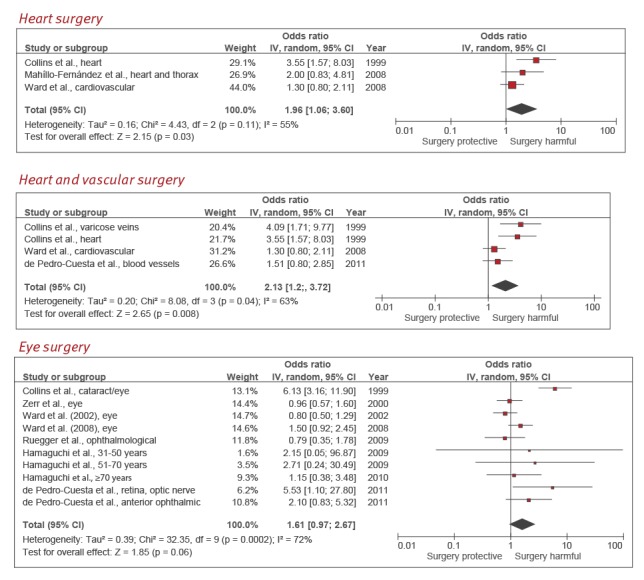
Forest plots of types of surgery positively related to sporadic Creutzfeldt–Jakob disease: heart surgery, heart and vascular surgery and eye surgery

CI: confidence interval; df: degrees of freedom; IV: interval variable.

We found three studies on the association between heart surgery and sCJD [18-20]. One of these included heart and thorax surgery [19] and another used the broad term cardiovascular surgery [20]. The summary odds ratio (OR) was 1.96 (95% confidence interval (CI): 1.06–3.60), with a high degree of heterogeneity (*I* ^2^ = 55%) (Figure 2).

All studies had a Newcastle–Ottawa scale score of at least 8 points, with the exception of one [18], which was also the only study not to use longitudinally sampled controls. A sensitivity analysis which excluded this latter study yielded a summary OR of 1.44 (95% CI: 0.94–2.19) with an *I* ^2^ = 0. The quality of evidence as defined by the GRADE rating system was very low (Table 2).

**Table 2:** GRADE evidence profile for case–control studies on the association between surgical procedures and incidence of Creutzfeldt–Jakob disease

Type of surgery	Risk of bias	Inconsistency (*I* ^2^)	Indirectness	Imprecision	Publication bias	High magnitude/ strength	Dose-response relationship	No residual confusion	Overall quality of evidence
Cardiovascular surgery	Yes	Yes (55%)	No	No	Not assessed	No	No	No	Very low
Vascular surgery	Yes	Yes (63%)	No	No	Suspected	No	No	No	Very low
Eye surgery	Yes	Yes (72%)	No	Yes	Suspected	No	No	No	Very low
Time between surgery and diagnosis >20 years	No	Not applicable	No	No	Not applicable	No	Yes	No	Not applicable
Appendectomy	Yes	No (38%)	No	No	Not assessed	No	No	No	Very low
Tonsillectomy	Yes	Yes (73%)	No	Yes	Not assessed	No	No	No	Very low
Dental surgery	Yes	Yes (72%)	No	Yes	Not assessed	No	No	No	Very low
Gall bladder	Yes	Yes (64%)	No	Yes	Not assessed	No	No	No	Very low
Neurosurgery	Yes	No (52%)	No	Yes	Not suspected	No	No	No	Very low
Any surgery	Yes	Yes (66%)	No	Yes	Not suspected	No	Yes	No	Very low

GRADE: Grading of Recommendations Assessment, Development and Evaluation [16].

**Heart and vascular surgery**

We found three papers with four case–control studies on the association between any vascular surgery, including heart surgery, and sCJD [18-21]. One paper addressed two different surgical exposures, heart and varicose vein surgery [18]. The summary OR was 2.13 (95% CI: 1.22–3.72), with a high degree of heterogeneity (*I* ^2^ = 63%; p = 0.04) (Figure 2). Two studies had a Newcastle–Ottawa scale score < 8, and controls were sampled at the end of a long study period rather than longitudinally [18]. On excluding these, the pooled OR was 1.37 (95% CI: 0.94–2.02). Despite the small number of studies, Egger’s test for publication bias showed a small p value of 0.09. The ‘trim and fill’ method to adjust for publication bias suggested a potential unpublished study with an OR of 0.76 (95% CI: 0.32–1.82). Its inclusion would yield a final summary OR estimate of 1.80 (95% CI: 1.04–3.08), though still with a high heterogeneity (*I* ^2^ = 66%). The quality of evidence as defined by the GRADE rating system was very low (Table 2).

**Eye surgery**

We found seven papers [18,20-25] which addressed 10 case–control studies. One paper divided subjects into three different age groups [24], whereas another divided eye surgery into two exposure groups, retina or optic nerve and anterior eye [21]. The summary OR was 1.61 (95% CI: 0.97–2.67), with a high degree of heterogeneity (*I* ^2^ = 72%; p = 0.0002) (Figure 2). Only in two studies was the Newcastle–Ottawa scale score > 7 [20,21], and the control sampling method was longitudinal in both. A sensitivity analysis restricted to the three case–control studies from these two papers yielded a summary OR of 1.87 (95% CI: 1.10–3.17), with a low degree of heterogeneity (*I* ^2^ = 19%). Although Egger’s test did not suggest publication bias (p = 0.90), the ‘trim and fill’ method suggested four potentially unpublished studies with point estimates that would have shown protective results for surgery. Inclusion of these four unpublished studies would give a summary OR estimate of 1.11 (95% CI: 0.62–1.97). The quality of evidence as defined by the GRADE rating system was very low (Table 2).

**Young age at surgery and a lapse longer than 20 years after surgery**

**Figure 3:**
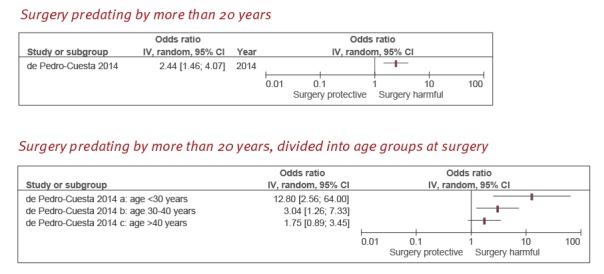
Odds ratio estimates of the association between history of surgery at least 20 years previously and sporadic Creutzfeldt–Jakob disease

CI: confidence interval; IV: interval variable.

We found only one case–control study on the association between age at surgery and sCJD in a population who had undergone surgery at least 20 years earlier [26]. In that study, there was a positive association between any major surgical procedure and sCJD, if surgery had occurred at least 20 years previously (OR = 2.44; 95% CI: 1.46–4.07) (Figure 3).

Overall estimate and estimates in three age groups. Data taken from [26].

This same study also showed that the OR for sCJD increased as age at surgery became younger, with evidence of a dose-response relationship and a strong effect measure in the youngest age group (Figure 3). Risk of bias was low because the Newcastle–Ottawa scale score was 10 and control sampling was longitudinal. In addition, it was the only study to have obtained data on surgical procedures from national discharge registries.

#### Types of surgery negatively related to sCJD

**Appendectomy**

**Figure 4:**
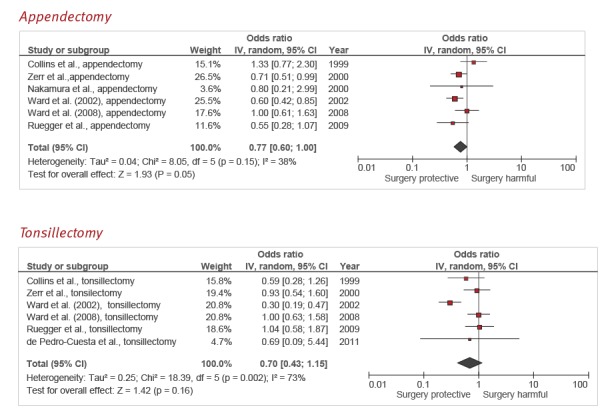
Forest plots of types of surgery negatively related to sporadic Creutzfeldt–Jakob disease: appendectomy; and tonsillectomy

CI: confidence interval; df: degrees of freedom; IV: interval variable.

There were six case–control studies that addressed the association between appendectomy and sCJD [18,20,22,23,25,27]. The summary OR was 0.77 (95% CI: 0.60–1.00) (Figure 4), with moderate heterogeneity (*I* ^2^ = 38%). Only one study had a Newcastle–Ottawa scale score > 7 [23] and its control sampling method was longitudinal. When the study with the highest OR estimate [18] was excluded, heterogeneity disappeared (*I* ^2^ = 0%) and the corresponding summary OR changed to 0.70 (95% CI: 0.57–0.85). GRADE quality of evidence was very low (Table 2).

**Tonsillectomy**

Six case–control studies addressed exposure to tonsillectomy in sCJD cases and non-CJD controls [18,20-23,25]. The summary OR was 0.70 (95% CI: 0.43–1.15), with a high degree of heterogeneity (*I* ^2^ = 73%; p = 0.002) (Figure 4). Only two studies had a Newcastle–Ottawa scale score > 7 [20,21] and in both, the control sampling method was longitudinal. The meta-analysis restricted to these two studies yielded a summary OR of 0.98 (95% CI: 0.63–1.54; *I* ^2^ = 0%). Leaving out the study with the most extreme results [23] made heterogeneity disappear and moved the summary estimate towards the null (pooled OR = 0.92; 95% CI: 0.69–1.21; *I* ^2^ = 0%). According to the GRADE rating system, the overall quality of evidence was very low (Table 2).

#### Types of surgery having no association with sCJD

**Dental surgery**

**Figure 5:**
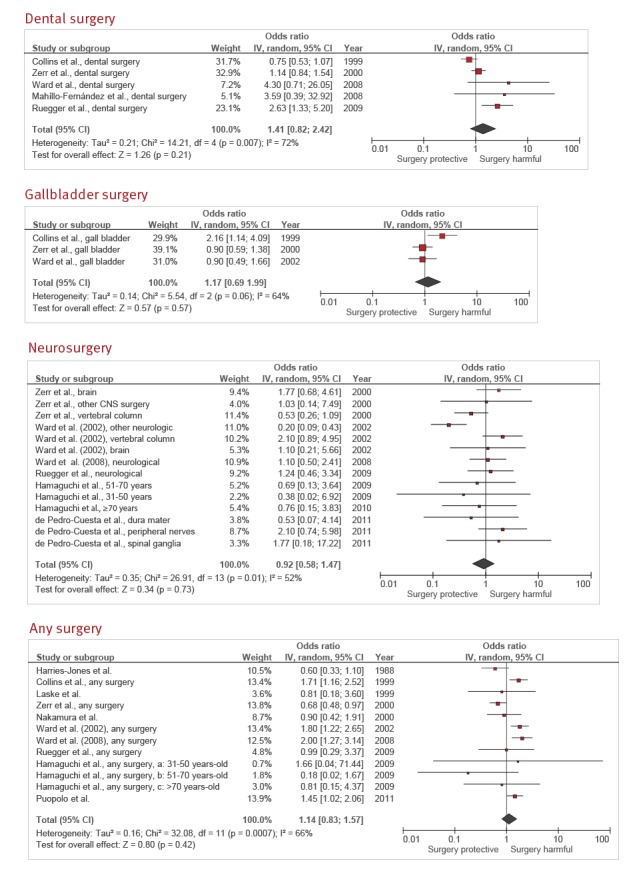
Forest plots of types of surgery having no association with sporadic Creutzfeldt–Jakob disease: dental surgery, gall bladder surgery, neurological surgery, and any surgery

CI: confidence interval; CNS: central nervous system; df: degrees of freedom; IV: interval variable.

Five case–control studies assessed the association between dental surgery and sCJD [18-20,22,25]. The summary OR estimate was 1.41 (95% CI: 0.82–2.42), with a high degree of heterogeneity (*I* ^2^ = 72%; p = 0.007) (Figure 5).

Two studies had a Newcastle–Ottawa scale score > 7 [19,20] as well as a longitudinal control sampling method. Nevertheless, these were the two studies that had least weight in the meta-analysis. A sensitivity analysis including only these two studies gave a summary pooled OR estimate of 4.00 (95% CI: 0.99–16.2; *I* ^2^ = 0%). The overall GRADE-rated quality of evidence was very low (Table 2).

**Gall bladder surgery**

Three case–control studies examined the association between gall bladder surgery and sCJD [18,22,23]. The summary OR was 1.17 (95% CI: 0.69–1.99), with a high degree of heterogeneity (*I* ^2^ = 64%; p = 0.06) (Figure 5). All three had a Newcastle–Ottawa scale score < 8 but only one [22] had implemented a longitudinal control sampling method. After excluding the latter study, heterogeneity disappeared (*I* ^2^ = 0%) and the new summary OR was 0.90 (95% CI: 0.69–1.99). The overall GRADE-rated quality of evidence was very low (Table 2).

**Neurosurgery**

There were six papers [20-25] which addressed the association between sCJD and surgical procedures in 14 different anatomical locations in brain and nervous system. The summary OR of neurosurgery for sCJD was 0.92 (95% CI: 0.58–1.47), with a high degree of heterogeneity (*I* ^2^ = 52%; p = 0.01) (Figure 5). Four case–control studies [20,21] scored > 7 on the Newcastle–Ottawa scale and all had used a longitudinal control sampling method. A sensitivity analysis restricted to these four studies yielded a summary OR estimate of 1.31 (95% CI: 0.73–2.33), with no heterogeneity (*I* ^2^ = 0%). Neither funnel plot nor Egger’s test nor the ‘trim and fill’ method suggested publication bias. The overall quality of evidence as defined by the GRADE rating system was very low (Table 2).

**Any surgical procedure**

Ten papers [18,20,22-25,27-30] studied the association between a history of any major surgery and sCJD in 12 case–control studies, where one paper [24] divided subjects into three age groups. The summary OR was 1.14 (95% CI: 0.83–1.57), with a high degree of heterogeneity (*I* ^2^ = 66%; p = 0.0007) (Figure 5). Only one study [24] had a Newcastle–Ottawa scale score > 7 and a longitudinal control sampling method. Publication bias was not suspected. The overall quality of evidence as rated by GRADE was very low (Table 2).

### Systematic review of case reports on potential transmission of sCJD by instruments used in neurosurgical procedures

**Figure 6:**
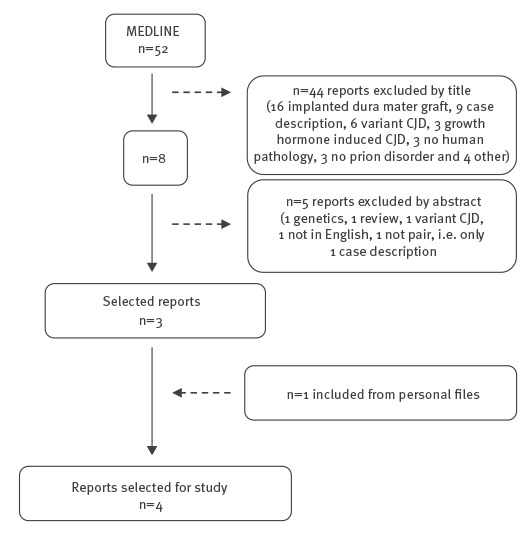
Flowchart of case reports of potential transmission of Creutzfeldt–Jakob disease by neurosurgery

**Figure 7:**
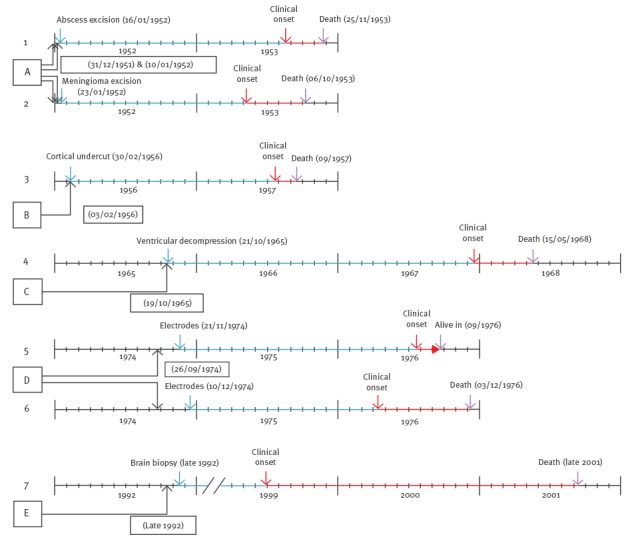
Temporal relationships between potentially infective procedures and the onset of Creutzfeldt–Jakob disease supposedly transmitted by neurosurgery (n = 7 cases)

Figure 6 shows the flowchart for the literature search. We identified four case reports [31-34] with seven sCJD cases potentially transmitted by neurosurgical procedures from five potential source patients. Figure 7 depicts the temporal relationships between potentially infective procedures and the onset of sCJD.

CJD: Creutzfeldt–Jakob disease.

Letters in squares: index cases; horizontal lines: potentially infected cases; blue lines: latency period between exposure and disease onset; red lines: period between disease onset and death or end of follow-up, whichever came first; arrows: specific dates. Case report references are [31] for A and B, [32] for C, [33] for D and [34] for E.

All incidents took place between January 1952 and late 1992, with a prolonged period from 1974 to 1992 that was free of reported cases. In all cases but one, direct contact between potentially reused surgical instruments and central nervous system was confirmed. In the case in which direct contact was not confirmed, a stereotactic brain biopsy had been conducted with a drill fitted with a disposable perforator [34]. Nevertheless, the exposure to reused instruments in this case was not as clear as in the other cases. Latencies between surgical procedures and sCJD onset ranged from 15.5 to 68 months (median: 19.5 months) and disease duration varied from 1.9 to 26 months (median: 5.4 months), although one patient was still alive two months after disease onset, at the time when the report was published.

## Discussion

This study sought to summarise the evidence on potential transmission of sCJD by surgical instruments. Our review of case–control studies suggests that vascular and eye surgery, together with surgery performed at very young age, might be associated with an increased risk of developing sCJD. It also suggests that tonsillectomy and appendectomy may not be associated with an increased risk of developing sCJD. Other types of surgery, such as dental, gall bladder, neurological and any surgical history, would not increase the risk. The overall quality of evidence was very low for all types of surgery. With regard to surgery at young age predating sCJD onset by more than 20 years, the strong positive findings reported by a recent study [26] have been neither confirmed nor rejected by any other study.

Our study has some limitations, and their effect on results may be difficult to assess. Since many studies were multipurpose in nature, interpretation needs to take into account possible bias frequently involved when studying surgical risk. When reviewing the included studies for potential biases, seven of 13 used hospital controls [22,24,25,27-30], seven had a nonsymmetrical exposure assessment (from close relatives in cases and directly from controls) [18,22-25,27,29], and in three studies, controls were sampled at the end of recruitment period for cases [18,23,25]. As a result, underestimation of risk towards the null was expected, particularly for recently introduced surgical techniques, such as coronary surgery. Such bias may act differentially across comparisons. Similarly, the high life-time risk of surgery in controls, close on 80% [20], and the fact that unexposed controls for a specific type of surgery might have been exposed to a different type of surgery [7], tend to bias the potential effect measure towards the null. Apart from one study [30], confounding by blood transfusion was hardly controlled for. The instrument used by us to quantify risk of bias in case–control studies, the Newcastle–Ottawa scale, does not capture all potential biases. On the other hand, we added control sampling method, which is very important in sCJD because controls should ideally be selected concurrently with cases (longitudinal sampling) to avoid selection bias. As we only searched studies in just one database, MEDLINE, we could have missed some studies. But a more comprehensive literature search we had conducted earlier found the same studies we found in MEDLINE [7]. Finally, the rating system used to assess quality of evidence, GRADE, penalises observational studies because it automatically rates them as having a lower quality of evidence than randomised controlled trials. Nonetheless, it reflects the fact that observational studies suffer from confounding and bias which may affect their internal validity [35]. Although the importance of observational studies was rated lower in our review, the experimentally tested infectivity of tissue from sCJD patients in non-human primates supports potential surgical transmission [5].

In all likelihood, the most challenging interpretation of our results pertains to neurosurgery. The negative result observed for neurosurgical history from case–control studies may still be consistent with moderate excess risk, masked by effect measures biased towards the null or with short latencies. Interestingly, clusters with two potential secondary cases, instead of pairs, were involved in two incidents. Strong support for disease transmission came from an incident where electrodes shared by three patients [33] were experimentally implanted into the cortex of a chimpanzee which developed the disease several years later [36]. Despite the fact that incidents related to the reuse of surgical instruments after neurosurgery on patients who were not known to have sCJD have been reported at CJD surveillance meetings or published since 1996 [37], only one case [34] has been described in a scientific journal in the past 40 years. As active CJD surveillance has been in place for the past 20 years, the few transmitted cases reported suggest that improvements in hygiene or preventive measures in neurosurgical operating theatres may have reduced sCJD transmission.

Our finding of the association between heart and vascular surgery and sCJD might be affected by potential bias towards a protective effect, suggesting that the association is a modest underestimate. However, meta-analysis restricted to those studies with less risk of bias yielded estimates closer to the null. The fact that the Danish and Swedish studies reported the risk period for coronary surgery [19,21] as that preceding sCJD onset by 10 years would support the contention that such surgical risk is a consequence of the disease, since a well-known vasculopathy and frequent stroke-like clinical onset have both been described [38]. Furthermore, the excess genetic risk of sCJD reported for APOE4, a vascular risk factor [39], would be consistent with an interpretation of surgical risk of sCJD as being a result of the disease itself, i.e. a result of its extracerebral manifestations.

Eye surgery is an infrequent surgery which is well studied and considered to be at special risk because of the traditional, though still uncertain, link between corneal transplantation and sCJD [3]. The summary measure in our meta-analysis, which provides a modest but precise excess risk, would be consistent with the only positive finding in the above register-based case–control study, i.e. an excess risk for retina surgery based on just three cases [21]. Assuming that, in all probability, the weight of retina surgery in eye surgery is modest, despite the expected bias towards the null, the excess risk observed by our study might be given some credibility.

The fact that the two surgical procedure groups with no evidence of association to sCJD, or even a potential protective role, corresponded to excision of lymphoid organs, appendectomy and tonsillectomy, is intriguing. Potential bias cannot be excluded since the papers with the most protective results were also those with the highest risk of bias. However, if their protective role were to be true, the fact that tonsillectomy corresponds to surgery at juvenile age, for which a specific susceptibility has been proposed, might suggest that reuse of surgical instruments restricted to a population free from CJD is safer. This finding might also suggest that the elimination of such organs, which could act as a possible prion entry point at some later date, could explain the modest beneficial effect.

History of surgery appears to indicate that there is no relationship between surgical history and sCJD or, at most, a modest excess risk mediated by bias.

The association between specific age at surgery and sCJD may warrant particular attention, since specific age at some exposures may have long-term health consequences. Examples for this are age at dietary exposure to bovine spongiform encephalopathy [40,41], age at first whooping cough and Parkinson’s disease [42], and an observed protective effect of a high educational level (acquired at juvenile age) on dementia or the risk of Parkinson’s disease from rural living and use of well water (most common in early decades of life) [43,44]. All these elements suggest the potential existence of a susceptibility to conformational neurodegenerative disorders which become clinically manifest many years later [39] and which might be related to the age at exposure. This hypothesis is supported by animal model research [45]. Recent findings of transmission of multiple system atrophy in cell and transgenic animal models [46], considered to be the second transmissible prion disease, and preliminary findings of surgical risk in other rapidly progressing neurodegenerative disorders such as amyotrophic lateral sclerosis, indicate the potential and the need for research in the field [45].

## Conclusion

The analyses conducted here on surgical risk of sCJD necessarily spanned a period of more than five decades. Given the considerable spread of study periods, the results of our study might reflect circumstances which change over time. In summary, our results show that: (i) the quality of evidence from analyses of surgical history and specific surgical procedure groups in case–control studies is very low, (ii) recent unreplicated studies with higher standards point to a high risk of surgery at low ages, with long time lags, and (iii) historical case reports suggest that transmission by neurosurgical instruments with a short time lag may occur.

Prevention of surgical transmission of sCJD relies on maintaining high standards in surgical procedures, defining high-risk situations (such as surgical management of mutation carriers) and implementing precautionary rules such as reserving, quarantining, destroying or incinerating potentially contaminated surgical instruments [8]. Such limited interventions are in contrast to the wide attention that hypothetical findings would receive that indicated a risk association between surgery and sCJD or other neurodegenerative disorders such as multiple system atrophy or amyotrophic lateral sclerosis [45].

The very low quality of evidence systematic reviews of case–control studies and in case reports of neurosurgical instruments cannot support any recommendation concerning the practice of surgery in relation to prevention of sCJD in the immediate future. However, if the incubation period between exposure and disease onset were to last several decades, one would not expect to find a surgical risk in most meta-analyses. Hence, too short a period between surgery and disease ascertainment in most case–control studies would account for our negative results. 

In view of the above results, it is unlikely that further studies on the association between general or specific types of surgery and sCJD will add much worthwhile information to what is already known, if the present low data quality, design problems and other sources of bias remain unchanged. In contrast, studies focused on the potential effect that surgery performed on young people has on sCJD incidence many years later, would probably shed light on the hypothesis that has received most support to date.
